# Pediatric Chronic Dialysis in Brazil: Epidemiology and Regional Inequalities

**DOI:** 10.1371/journal.pone.0135649

**Published:** 2015-08-18

**Authors:** Tulio Konstantyner, Ricardo Sesso, Maria Fernanda de Camargo, Luciana de Santis Feltran, Paulo Cesar Koch-Nogueira

**Affiliations:** 1 Sociedade Hospital Samaritano, Sao Paulo, Brazil; 2 Pediatric Division, Federal Univesity of Sao Paulo, Sao Paulo, Brazil; 3 Nephrology Division, Federal University of Sao Paulo, Sao Paulo, Brazil; Universidade de Sao Paulo, BRAZIL

## Abstract

**Introduction:**

There are few reports in the literature estimating the epidemiologic characteristics of pediatric chronic dialysis. These patients have impaired physical growth, high number of comorbidities and great need for continuous attention of specialized services with high demand for complex and costly procedures.

**Objective:**

The aim of this study was to estimate the incidence and prevalence rates and describe the characteristics of children and adolescents undergoing chronic dialysis treatment in a Brazilian demographic health survey.

**Materials and Methods:**

A cross-sectional study was performed in a representative sample of dialysis centers (n^c^ = 239) that was established from the 2011 Brazilian Nephrology Society Census (N^c^ = 708). We collected data encompassing the five Brazilian macro-regions. We analyzed the data from all patients under 19 years of age. The sample population consisted of 643 children and adolescents who were on chronic dialysis program anytime in 2012. Data collection was carried out in the dialysis services by means of patients' records reviews and personal interviews with the centers’ leaders.

**Results:**

We estimated that there were a total of 1,283 pediatric patients on chronic dialysis treatment in Brazil, resulting in a prevalence of 20.0 cases per million age-related population (pmarp) (95% CI: 14.8–25.3) and an incidence of 6.6 cases pmarp in 2012 (95% CI: 4.8–8.4). The South region had the highest prevalence and incidence rates of patients under dialysis therapy, 27.7 (95% CI: 7.3–48.1) and 11.0 (95% CI: 2.8–19.3) cases pmarp, respectively; the lowest prevalence and incidence rates were found in the North-Midwest region, 13.8 (95% CI: 6.2–21.4), and in the Northeast region, 3.8 (95% CI: 1.4–6.3) cases pmarp, respectively.

**Conclusion:**

Brazil has an overall low prevalence of children on chronic dialysis treatment, figuring near the rates from others countries with same socioeconomic profile. There are substantial differences among regions related to pediatric chronic dialysis treatment. Joint strategies aiming to reduce inequities and improving access to treatment and adequacy of services across the Brazilian regions are necessary to provide an appropriate care setting for this population group.

## Introduction

The pediatric end-stage renal disease (ESRD) is characterized by the severe irreversible kidney damage and the reduction in glomerular filtration rate (GFR) to less than 15 ml/min/1.73 m^2^. It has been recognized as a major public health problem due to the growing number of children receiving renal replacement therapy (RRT) worldwide (dialysis or kidney transplantation), providing evidence of improved long-term survival of children with chronic kidney disease (CKD) who progress to advanced stage of the disease [1].

Chronic kidney disease in children is a devastating illness and the mortality rate for children with ESRD receiving dialysis therapy is between 30 and 150 times that of the general pediatric population [2]. Moreover, the substantial number of children with ESRD without access to RRT shows the need to both develop low-cost treatments and implement effective population-based prevention strategies [3].

There are marked disparities in the incidence of treated ESRD worldwide [4, 5]. Whether these disparities reflect differences in risk factors and prevalence of CKD among continents has been poorly investigated, particularly in children and adolescents. There are few reports in the literature estimating the epidemiologic characteristics of chronic dialysis treatment in this age group, which can vary in different geographical regions probably as a result of racial and ethnic distribution, prevalent types of kidney diseases and quality of health care provided. Furthermore, there is evidence of changes in this profile due to the advance of therapy and the gradual increasing number of treated individuals [6, 7].

In 2011, the report of Brazilian census of chronic dialysis by the Brazilian Society of Nephrology pointed over 1,400 children on chronic dialysis program and a recent observational study conducted in the state of Sao Paulo found a prevalence of 23.4 cases per million age-related population (pmarp). Both surveys reported demographic data and information on underlying renal disease, but did not investigate the regional characteristics, treatment approaches and factors associated with pediatric patients on chronic dialysis treatment [8, 9].

Since Brazil is a developing country with a large territorial area and great socioeconomic and cultural diversity, the regional differences can express peculiar epidemiological characteristics of those patients. Such knowledge can guide strategies for control and prevention of chronic kidney failure, aiding early diagnosis, the effectiveness of reducing the rate of progression of renal disease and treatment planning for such a disorder, especially, health managers from other countries with similar demography [10, 11].

The aim of this study was to describe the epidemiological characteristics and estimate the incidence and prevalence rates of pediatric cases undergoing chronic dialysis treatment in a Brazilian demographic health survey.

## Materials and Methods

### Study design

The present investigation is a cross-sectional study of a stratified random sample of Brazilian dialysis centers. For over 10 years, the Brazilian Society of Nephrology (BSN) has organized an annual national census of patients on chronic dialysis treatment. The 2011 census, comprising of 708 centers, was used to collect the names and locations of dialysis centers [12].

### Sample Plan of Dialysis Centers

We selected a stratified random sample with proportional distribution of dialysis centers that included the five Brazilian macro-regions (North, Midwest, Northeast, Southeast and South).

Since the distribution of dialysis centers in Brazil was not uniform, the North and Midwest macro-regions were considered as one region to have a minimum of 30 dialysis centers from each region. This was the required number to implement the sampling plan that considered the dialysis centers as primary sampling units and was a prerequisite for a more dependable estimation of the incidence and prevalence rates of pediatric cases undergoing chronic dialysis treatment [13].

The selection of the dialysis centers to form the sample was done by stratification into three groups of patients up to 19 years of age in each center, according to the 2011 BNS census as follows: a) centers with five or more patients (group 1—N^c^ = 35), b) centers with four or less (group 2—N^c^ = 363), and c) centers with unknown number of patients due to failure to respond to the 2011 census (group 3—N^c^ = 310). The dialysis centers were proportionally distributed in four macro-regions adopted in the sampling plan (North plus Midwest, Northeast, Southeast and South).

The calculation of the sample size of dialysis centers was based on the objective of estimating the total number of children and adolescents undergoing dialysis in Brazil.

All dialysis centers from group 1 were included in the studied sample. For groups 2 and 3, the sample size (number of dialysis centers) was calculated using the equation $n=\frac{C^{2}}{{CV}^{2}}$, where, $C^{2}=\frac{S^{2}}{{\bar{Y}}^{2}}$ is the coefficient of variation per center; $\bar{Y}$ is the average number of children/adolescents who underwent chronic dialysis per center; *S* ^*2*^ is the variance of the number of children/adolescents per center; and *CV* ^2^ is the coefficient of variation tolerated (0.10). According to the data obtained in the 2011 BSN Census, $\bar{Y}$ = 0.73 and *S* = 1.04.

With these assumptions the estimated number of centers in groups 2 and 3 was 202 (n = 101 in each one). In both groups the centers were proportionally distributed and sorted systematically by region. They were selected through a random selection of the centers identified in the 2011 Census (simple random sampling of each region of Brazil). To compensate for different selection probabilities used in each group, weights defined by the inverse of the sampling fraction were used [13].

Thus, overall, we estimated the need to visit at least 237 dialysis centers in Brazil to meet the objective of the study: 35 in group 1, 101 in group 2 and 101 in group 3.

As we expected 5% of services were inactive and in 15% of randomly selected centers the necessary information would not be given, we included an extra 20% of centers in groups 2 and 3 to compensate for non-response. Thus, 293 dialyses centers obtained by probability sampling were contacted.

After telephone contact with the technical leaders of these centers, 43 were excluded from the study for the following reasons: 32 did not treat children or adolescents, 7 discontinued to function in 2012 and 4 due to address change. A research team including nine graduate health professionals selected for field activities visited 250 dialysis centers.

**Fig 1:**
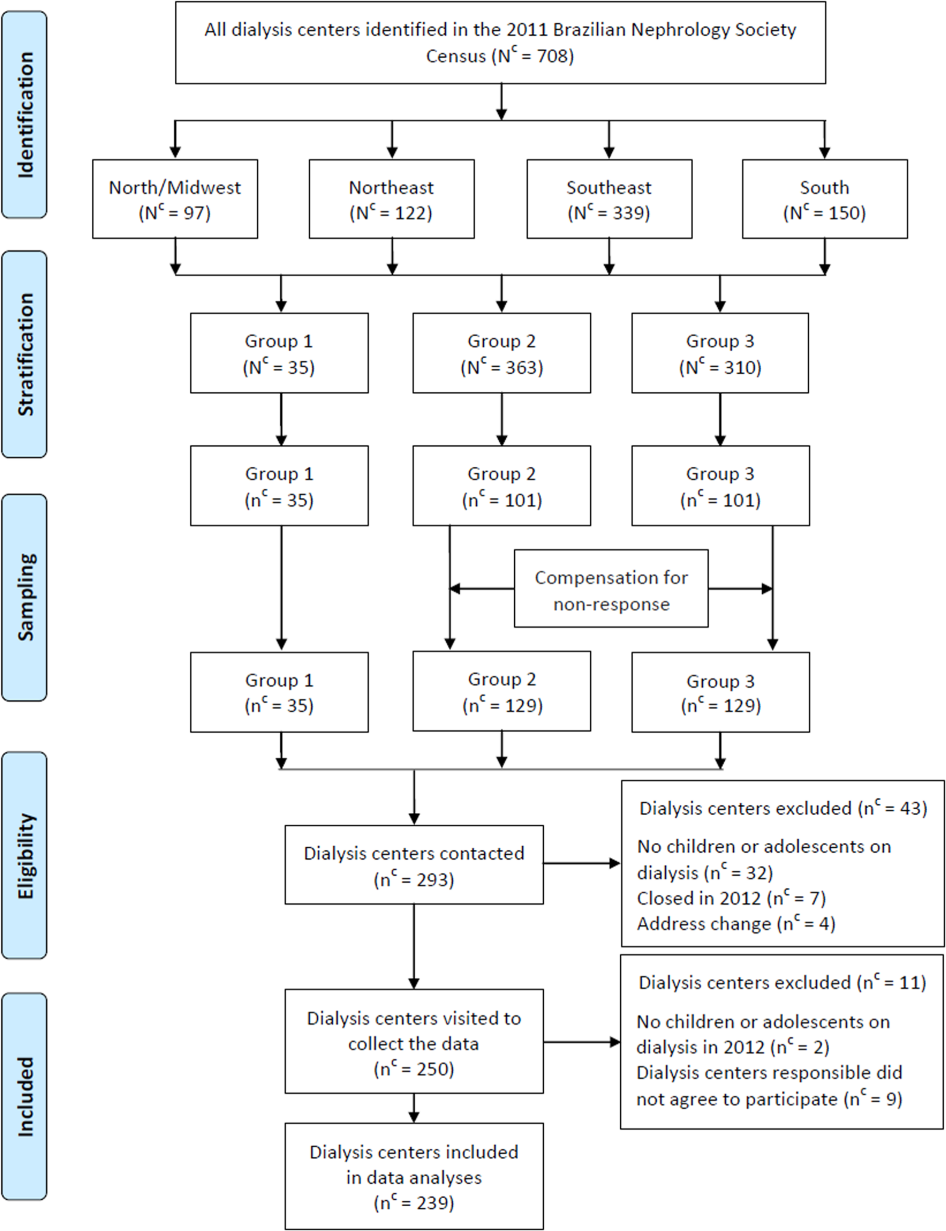
Flow diagram of the sampling process of chronic dialysis centers, Brazil, 2012. N^c^: Total number of dialysis centers in Brazil, macro-regions and groups; n^c^: number of dialysis centers in the sample; Group 1: centers with five or more patients; Group 2: centers with four or less patients; Group 3: centers with unknown number of patients.

After visiting the centers, we further excluded 11 from the study due to: 2 not treating children or adolescents in 2012 and 9 that did not agree to participate and did not sign the term of consent. Therefore, data from 239 dialysis centers were used to represent the situation in the country. The sampling process of dialysis units is shown in a flow diagram (Fig 1).

### Sample Population

**Fig 2:**
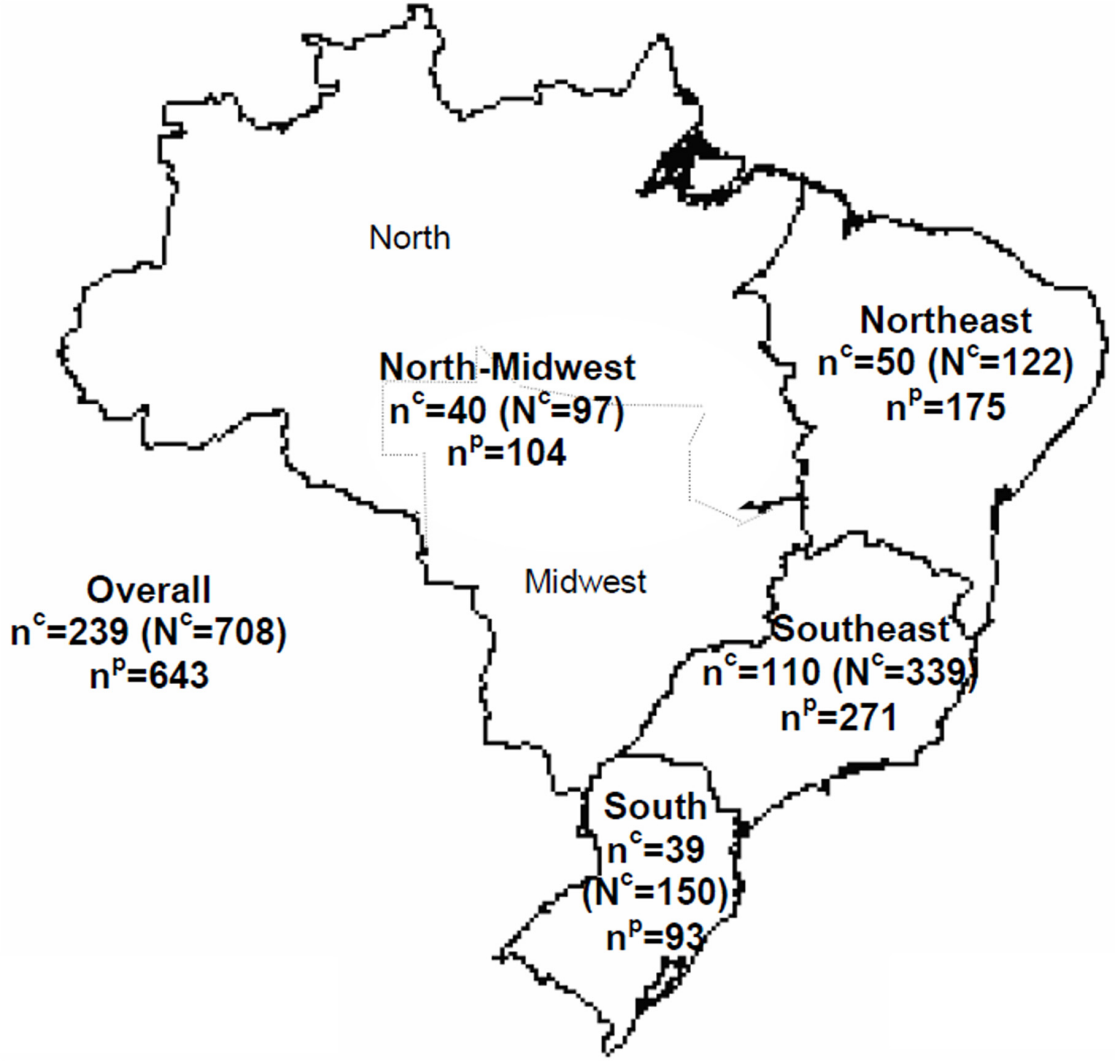
Distribution of number of chronic dialysis centers included in the sample, total number of dialysis centers, and number of patients participating in the survey in Brazil, by region, 2012. Number of dialysis centers (DC) selected (n^c^) and total number of dialysis centers in Brazil and per region (N^c^—in parenthesis); n^p^: sample population—children and adolescents who were on chronic dialysis program.

We collected and analyzed the data from all patients under 19 years of age with ESRD who attended the included dialysis centers. The sample population consisted of 643 children and adolescents who were included in a chronic dialysis program anytime in 2012 (Fig 2).

### Data Collection

A structured and pre-encoded questionnaire was used for data collection from the health records of children and adolescents, including demographic, clinical and epidemiological variables: age group, gender, race, type of dialysis (hemodialysis or peritoneal dialysis), source of dialysis reimbursement (public or private), type and age at primary diagnosis, age at beginning of dialysis therapy and history of previous kidney transplantation. Interviewers were trained and given a set of norms and definitions to fill in the questionnaire [14].

Data was collected at the dialysis centers from both patients’ records reviews and personal interviews with the dialysis leader, usually a physician or nurse. All procedures were standardised and tested in the pre-test stage of the project by an interdisciplinary field team.

### Statistical Analysis

The completed questionnaire was evaluated in terms of its internal consistency prior to data entry for analyses. The information was transcribed in databases using double entry and posterior validation in order to correct errors. The Stata statistical software package Version 13.0 (College Station, TX: StataCorp LP) was used to analyse the data and expand the sample by using the module "svy", which allows considering the different aspects of the complex sampling design [15].

To provide a countrywide pediatric ESRD profile, we estimated the rates of incidence and prevalence of children and adolescents undergoing chronic dialysis treatment in 2012. The incidence rate was defined as the ratio of the number of new cases of patients up to 19 years receiving chronic dialysis program during 2012 divided by the total existing population under 19 years of age in Brazil and macro-regions, in July 1st, 2012. The prevalence rate was defined as the ratio of the number of all cases (new and old) of the same kind of patients during the same year divided by the same total population [16].

Particularly, we estimated the average age when the primary diagnosis was established and the average time in years between primary diagnosis and initiation of chronic dialysis program in Brazil and regions, using just the subjects who had such data available.

Data consistency analysis and univariate descriptive statistics were performed for continuous and categorical variables. In the case of comparison of categorical variables, the Pearson's chi-square test was used. A maximum level of p = 0.05 was chosen to indicate a significant association [17].

All statistical procedures were performed with the sample expansion technique, taking into account the sampling design, which provided national representativeness for the studied macro-regions of Brazil (North plus Midwest, Northeast, Southeast and South). To estimate the total number of children, the weighting and stratification (groups of dialysis centers) of sampling units were considered. Additionally, to obtain estimates of data on characteristics of children, it was still considered the drawing of clusters, since the dialysis centers have become the primary sampling units drawn in single stage.

### Ethical Statement

The technical leaders of dialysis centers signed terms of informed consent for data use. The data of the children and adolescents were collected from the health records. There was no direct contact with the study subjects or any personal identification. The confidentiality of information was preserved. The Ethics and Research Committee of the 'Hospital Samaritano de São Paulo' approved this consent procedure and also the fulfilment of the present study (n° 27450814.9.0000.5487).

## Results

We estimate that there were a total of 1,283 pediatric patients with ESRD on chronic dialysis treatment in Brazil. The average age was 12.5 years (95% CI: 11.7–13.4) and the distribution of races was 45.8% Caucasians, 42.4% mulattos, 0.2% indigenous, 11.1% blacks and 0.6% others.

The overall incidence of children and adolescents with ESRD on dialysis treatment was 6.6 cases pmarp in 2012 (95% CI: 4.8–8.4). The South region stood out with the highest rate of new pediatric cases under such therapy: 11.0 cases pmarp (95% CI: 2.8–19.3). The Northeast region had the lowest rate, 3.8 (95% CI: 1.4–6.3) (Table 1).

**Table 1:** Absolute numbers, incidence and prevalence rates of cases of pediatric end-stage renal disease on chronic dialysis treatment—Brazil and regions, 2012.

Geographic area	I	(95% CI)	N (%)	P	(95% CI)	N (%)
**Brazil**	6.6	(4.8–8.4)	424 (100)	20.0	(14.8–25.3)	1283 (100)
North-Midwest	5.5	(2.2–8.8)	64 (15.1)	13.8	(6.2–21.4)	161 (12.6)
Northeast	3.8	(1.4–6.3)	75 (17.7)	16.4	(4.6–28.1)	319 (24.9)
Southeast	7.9	(4.3–11.5)	192 (45.3)	23.3	(14.3–32.3)	569 (44.3)
South	11.1	(2.8–19.3)	93 (21.9)	27.7	(7.3–48.1)	234 (18.2)

I: Annual incidence per million aged compatible population (2012) P: Prevalence per million aged compatible population; CI: Confidence Interval; N: Absolute number of cases.

During 2012, the prevalence of pediatric ESRD on chronic dialysis treatment was 20.0 cases pmarp (95% CI: 14.8–25.3). Once more, the South region had the highest prevalence rate of patients under therapy: 27.7 cases pmarp (95% CI: 7.3–48.1). The North-Midwest region had the lowest rate, 13.8 (95% CI: 6.2–21.4) (Table 1).

Table 2 shows the prevalence of the characteristics of pediatric patients in Brazil, comparing the four macro-regions. The proportion of male gender was 52.5% and female was 47.5%. The prevalence of adolescents (64.8%, 95% CI: 56.9–71.9) was significantly higher than children (35.2%, 95% CI: 28.1–43.1), and hemodialysis (HD) was proportionally more used than peritoneal dialysis (74.9%, 95% CI: 66.9–81.6, vs 25.1%, 95% CI: 18.4–33.1, respectively). The proportion of patients who underwent previous kidney transplantation was 23.1% (95% CI: 17.8–29.4).

**Table 2:** Prevalence of the characteristics of patients under 19 years of age undergoing chronic ambulatory dialysis program—Brazil and regions, 2012 (np = 1283).

Characteristics of patients	Overall	Geographic area				p-value
		North-Midwest	Northeast	Southeast	South	
**Age group**						0.934
Children	35.2 (28.1–43.1)	31.4 (18.5–47.9)	38.3 (21.9–57.8)	35.5 (26.6–45.6)	32.9 (16.9–54.1)	
Adolescents	64.8 (56.9–71.9)	68.6 (52.1–81.5)	61.7 (42.2–78.1)	64.5 (54.4–73.4)	67.1 (45.9–83.1)	
**Gender**						0.355
Male	52.5 (48.5–56.5)	54.2 (45.1–63.0)	52.2 (46.8–57.7)	55.5 (49.3–61.7)	44.9 (31.5–59.1)	
Female	47.5 (43.5–51.5)	45.8 (37.0–54.9)	47.8 (42.3–53.2)	44.5 (38.3–50.7)	55.1 (40.9–68.5)	
**Race**						<0.001
Caucasian	45.8 (36.2–55.7)	14.3 (7.8–24.7)	23.6 (14.4–36.2)	50.8 (41.0–60.6)	87.9 (78.0–93.8)	
Non Caucasian	54.2 (44.3–63.8)	85.7 (75.3–92.2)	76.4 (63.8–85.6)	49.2 (39.4–59.0)	12.1 (6.2–22.0)	
**Type of dialysis**						0.811
Hemodialysis	74.9 (66.9–81.6)	76.3 (49.1–91.5)	74.1 (62.3–83.2)	78.0 (65.2–87.0)	68.4 (45.9–84.6)	
Peritoneal^a^	25.1 (18.4–33.1)	23.7 (8.5–50.9)	25.9 (16.8–37.7)	22.0 (13.0–34.8)	31.6 (15.4–54.1)	
**Health system** ^**b**^						0.075
Public	90.5 (85.5–93.9)	95.7 (86.7–98.7)	96.2 (89.3–98.7)	86.7 (78.6–92.1)	86.8 (71.6–94.5)	
Private	9.5 (6.1–14.5)	4.3 (1.3–13.3)	3.8 (1.3–10.7)	13.3 (7.9–21.4)	13.2 (5.5–28.4)	
**Primary diagnosis**						0.011
Known	67.7 (60.3–74.3)	54.2 (44.7–63.3)	53.7 (34.4–71.9)	78.9 (70.0–85.7)	72.0 (58.6–82.3)	
Unknown	32.3 (25.7–39.7)	45.8 (36.7–55.3)	46.3 (28.1–65.6)	21.1 (14.3–30.0)	28.0 (17.7–41.4)	
**Age at dialysis beginning**						0.834
<12 years	44.9 (36.8–53.3)	38.8 (26.4–53.0)	47.8 (28.2–68.1)	46.9 (36.6–57.5)	40.3 (22.2–61.5)	
>12 years	55.1 (46.7–63.2)	61.2 (47.0–73.6)	52.2 (31.9–71.8)	53.1 (42.5–63.4)	59.7 (38.5–77.8)	
**Previous kidney transplantation**						0.014
Yes	23.1 (17.8–29.4)	8.7 (2.2–28.9)	18.2 (9.6–32.0)	22.8 (16.0–31.3)	40.3 (29.3–52.5)	
No	76.9 (70.6–82.2)	91.3 (71.1–97.8)	81.8 (68.0–90.4)	77.2 (68.7–84.0)	59.7 (47.5–70.7)	

Values are percent prevalence (95% confidence interval).

^a^Continuous ambulatory peritoneal dialysis (10.4%), intermittent peritoneal dialysis (5.6%) or automated peritoneal dialysis (84%).

^b^Source of dialysis reimbursement.

The comparison among regions showed that the Southeast had the highest proportion of known primary diagnosis (78.9%, 95% CI: 70.0–85.7, p = 0.011) and the South region had the highest proportion of Caucasians (87.9%, 95% CI: 78.0–93.8, p<0.001) and of patients who received previous kidney transplantation (40.3%, 95% CI: 29.3–52.5, p = 0.014).

**Fig 3:**
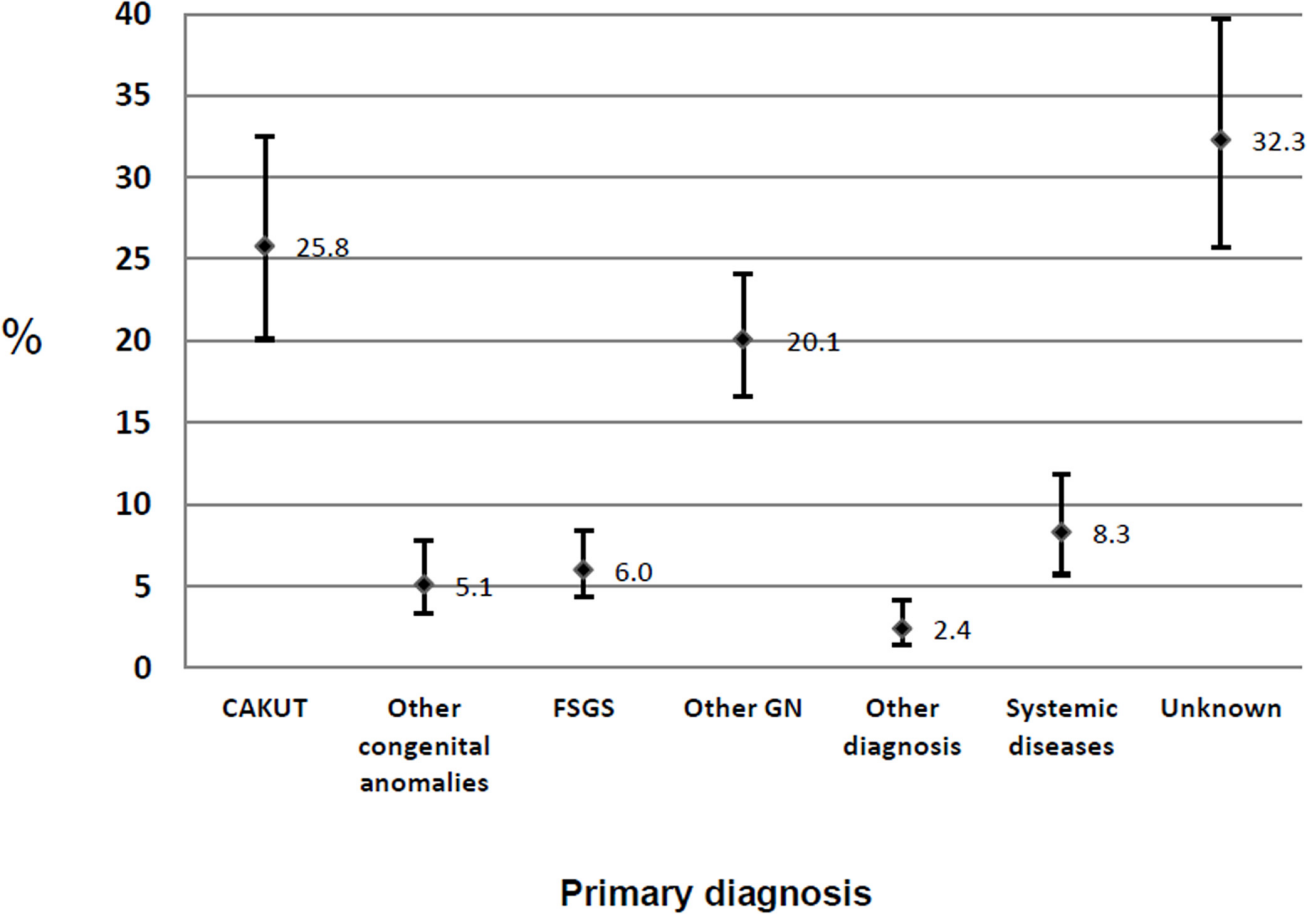
Prevalence of primary diagnosis in patients under 19 years of age with CKD and kidney failure undergoing chronic ambulatory dialysis—Brazil, 2012 (n^p^ = 1283). CAKUT: Congenital anomalies of kidney and urinary tract; FSGS: Focal Segmental Glomerulosclerosis; GN: Glomerulonephritis; Values are prevalence and 95% confidence interval.

Fig 3 shows the prevalence of primary diagnosis in subjects undergoing chronic dialysis program. Overall the highest rate was related to unknown diagnosis (32.3%, 95% CI: 25.7–39.7) and the main known diagnosis was related to congenital anomalies of kidney and urinary tract (CAKUT) with a prevalence of 25.8% (CI 95% CI: 20.1–32.5).

**Fig 4:**
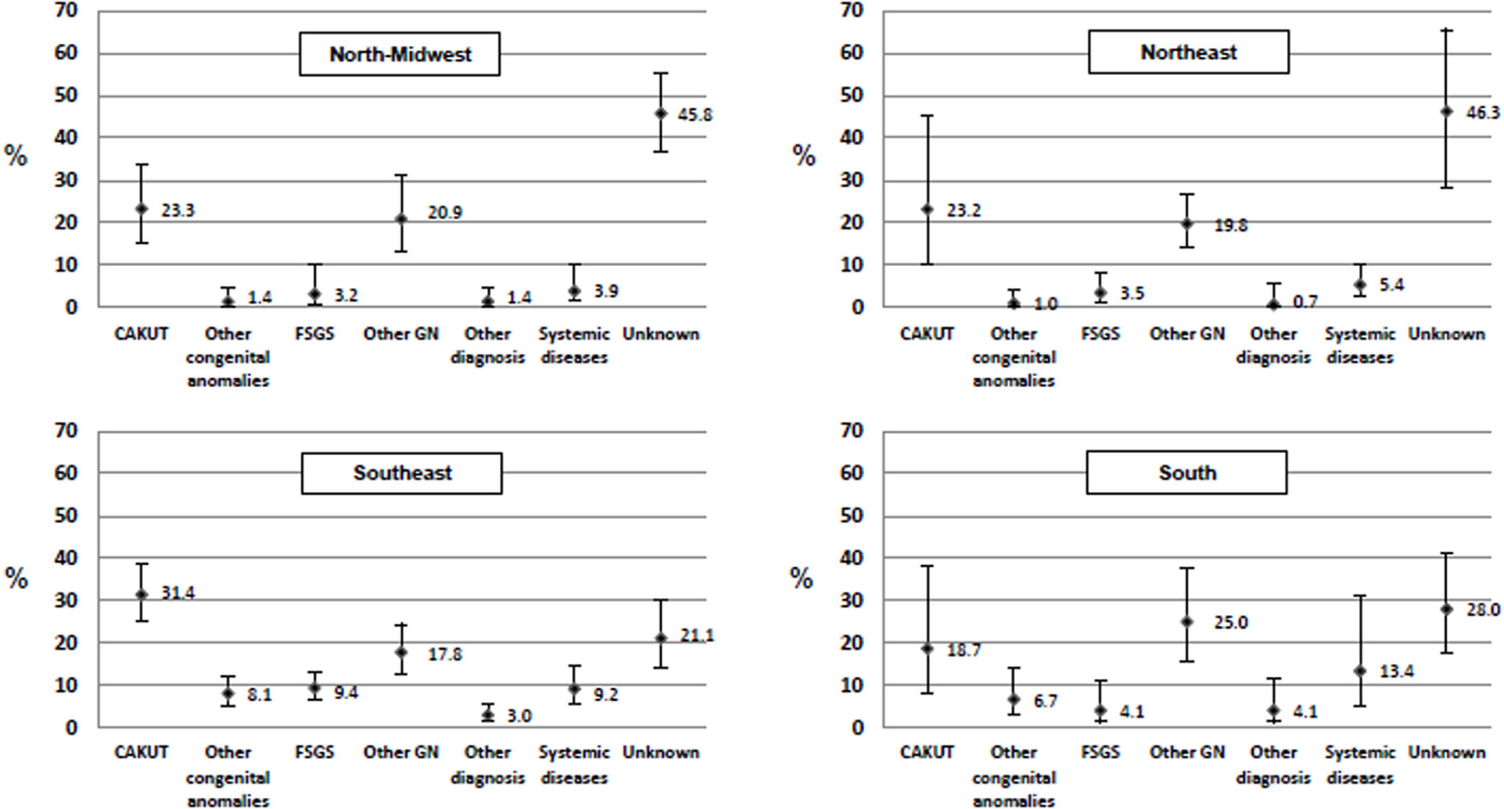
Prevalence of primary diagnosis in patients under 19 years of age with CKD and kidney failure undergoing chronic ambulatory dialysis—regions of Brazil, 2012. CAKUT:Congenital anomalies of kidney and urinary tract; FSGS:Focal Segmental Glomerulosclerosis; GN:Glomerulonephritis; Values are shown as percent and 95% confidence interval; P value for the comparison of primary diagnosis prevalence among the four macro-regions, p = 0.025. Number of patients estimated per region, North-Midwest n^p^ = 161, Northeast n^p^ = 319, Southeast n^p^ = 569, South n^p^ = 234.

Fig 4 shows the comparison of such prevalence among the four regions. The prevalence of CAKUT was relatively higher in the Southeast region (31.4%, 95% IC: 25.0–38.6) followed by the North-Midwest region (23.3%, 95% IC: 15.3–33.9) and Northeast region (23.2%, 95% IC: 10.0–45.2); and lower in the South region (18.7%, 95% IC: 7.9–38.2).

The overall average patients' age at primary diagnosis was 8.3 years (95%CI: 6.6–10.0), and 9.1 years (95% CI: 6.5–11.6) in the North-Midwest region, 10.2 years (CI 95% 8.1–12.4) in the Northeast, 6.8 years (CI 95% 4.2–9.4) in the Southeast and 11.4 years (CI 95% 6.6–16.2) in the South region.

The median time between diagnosis of primary renal disease and initiation of chronic dialysis program, in a subsample of 375 children who had information about their age at the time of primary diagnosis, was greater in the Southeast region (14.5 months, IQR: 0.2–115.8), while this value was 0.9 months (IQR 0.0–2.8) in the North-Midwest region, 0.0 months (IQR 0.0–0.0) in the Northeast region and 0.0 months (IQR 0.0–1.0) in the South region (overall p-value < 0.001).

## Discussion

The results of this study indicate that the incidence and prevalence of pediatric ESRD on chronic dialysis treatment in Brazil in 2012 were approximately 6 and 20 cases pmarp, respectively. The highest rates occurred in regions South and Southeast while the lowest in regions North-Midwest and Northeast. The prevalence rates of three characteristics of the children were significantly different among regions: race, primary diagnosis and previous kidney transplantation.

In contrast to the adult population, in whom registries usually have confirmed the incidence, prevalence, and primary diagnoses of CKD, the epidemiological information on pediatric CKD has been pointed as imprecise and flawed by methodological differences among various data sources [1].

The number of incident ESRD cases per year among children has been generally stable for the past two decades in the United States (US) [18]. However, the incidence of RRT in pediatric patients in Europe from 2009 to 2011 has varied markedly between countries [5]. A recent review of epidemiology of CKD in children showed that the median incidence rate of RRT in children aged less than 20 years worldwide was around 9 pmarp in 2008, varying from less than 6 (Russia, Finland, Japan and Switzerland) to more than 15 pmarp (United States, Scotland and New Zealand) [19].

The present study shows an incidence of pediatric ESRD on chronic dialysis treatment in Brazil similar to the lowest incidences reported by the review. It is possible that the ethnicity of Brazilian children is less predisposed to acquire a primary disease that causes ESRD. However, it is more likely that the low incidence is associated with the great socioeconomic diversity and the development level of country regions, which can favor the underdiagnosis of CKD [19].

At the same time, the number of prevalent ESRD patients continues to increase in all age groups in the US [18]. The prevalence of children and adolescents on RRT ranged from 18 (Russia) to 100 pmarp (Finland) in 2008. Such oscillation has been associated with the quality and the development of health systems from countries [19].

We found a low prevalence of ESRD children and adolescents on chronic dialysis treatment in Brazil, figuring near of rates from others countries with same socioeconomic profile. Sometimes, developing countries do not accomplish the early diagnosis of CKD, remaining blind to identification of sick children, and cannot afford or prioritize the costs of RRT, decreasing the survival of children with ESRD [19, 20].

Approximately 80% of RRT patients worldwide live in Europe, Japan or North America, where all children with ESRD have access to RRT [19]. Although Brazil has a low prevalence rate, the absolute number of children in RRT found in the country is one of the largest ones, which places Brazil in a prominent position in the world with regard to absolute number of children in dialysis.

Nevertheless, it is important to recognize that there are extensive differences among Brazilian regions. For instance, the Interagency Network of Health Information (RIPSA), a national database created in 1996 jointly by the Ministry of Health of Brazil and Pan American Health Organization (PAHO), showed that health and socioeconomic indicators have been constantly better in the South and Southeast regions, when comparing with the North, Northeast and Mid-West [21].

Moreover, the United Nations Development Program published the Atlas of Human Development in Brazil, which reported that Southeast and South regions had the best averages of Municipal Human Development Index in 2010 (MHDI-2010) [22]. The availability of resources to health strategies is substantially higher in the Southeast, the main socioeconomic region, and in the South, the region showing the lowest child mortality rate. That is the most likely explanation on the differences of prevalence and incidence rates among regions found in the present research. Table 3 presents the main socioeconomic and health indicators of Brazil and the four geographical regions compared [21, 22].

**Table 3:** Socioeconomic and health indicators of Brazil and regions [21, 22].

Socioeconomic and health Indicators	Overall	Geographic area			
		North/ Midwest	Northeast	Southeast	South
Total Population^a^	193.98	30.77	53.91	81.57	27.73
Population under 19 years^a^	64.02	11.63	19.54	24.41	8.44
Population density (people per km^2^)	22	6	34	87	49
Child Mortality Rate^b^	15.3	17.7	18.0	13.0	11.0
Gross Domestic Product (US$)^c^	11,596	9,712	4,987	14,119	11,185
Health investment (US$)^c^	403	341	311	448	388
Human Development Index (2010)^d^	0.730	0,705	0.660	0.754	0.756
Life expectancy at birth (years)	74.5	72.8	71.9	76.2	76.5
Illiteracy rate (2010)^e^	9.4	9.4	18.5	5.3	5.0

^a^Millions of inhabitants

^b^Number of infant deaths (under 1 year) per 1,000 live births

^c^ *Per capita* in 2012

^d^Average Human Development Index of Federation States in 2010

^e^Percentage of the population illiterate in 2010 (15 years of age or more).

Although there are centers specialized in RRT in all regions in Brazil, patient families often migrate from less developed socioeconomic areas to regions with better indices of human development looking for adequate treatment. In fact, Southeast region figures as the largest one receiving children with CKD from all other Brazilian States [23].

Although rates of the South and Southeast regions have been higher than others regions, the prevalence rates are still low compared with those from developed countries. Such evidence raises the need to improve actions to identify early causes of CKD and the access to RRT to all pediatric patients with ESRD in the entire country.

The prevalence of adolescents with ESRD on chronic dialysis treatment was significantly higher than children. That difference occurred similarly in all regions and it is also reported in other studies using samples of patients with ESRD [5]. The explanation might be partly related to the timing of initiation of RRT and the primary diagnosis of CKD [19]. Indeed, more than a half of our sample had the dialysis program beginning after 12 years of age.

We observed a discrete preponderance of male over female children. This finding is traditionally attributed to the higher frequency of obstructive CAKUT in boys and is aligned with the literature [8, 11, 19, 20].

This study found an overall proportion of Caucasian children slightly lower than the other races. This racial distribution differs somewhat from the distribution reported in studies on other continents, which showed higher proportions of African-American, indigenous and South Asian with ERSD than white children [8, 18, 19, 23]. This contradiction may be related to access to health services, for the white population in Brazil generally live under better socioeconomic conditions. Although lack of equity determined by the race is a relatively unexplored subject in the healthcare literature in Brazil, social inequalities determine the black people biological vulnerability [24].

Comparing Brazilian regions, South region had the highest proportion of Caucasians children on RRT. Such significant difference is contradictory to the expected result because it was the region with the highest incidence and prevalence of RRT in Brazil. This finding may be associated with racial characteristic of this region that received over the past decades a large contingent of European immigrants [25].

Hemodialysis was the main treatment modality, being used by three quarters of the patients. Countries vary considerably in the distribution of initial treatment modality [26]. Renal replacement therapy varies with age since peritoneal dialysis (PD) is the preferred choice in younger children in Europe and the United States [18, 27]. Adolescents are treated primarily by HD, because the management of many of them is in adult dialysis units where HD is more likely to be proposed than in pediatric units. This scenario is the same as happens in Brazil [8].

Brazil has a unique public health system ('Sistema Único de Saúde'—SUS), which is based on the principles of integral health as a right of citizens and a duty of the State. The public health system was the main source of dialysis reimbursement in all regions in our study. Nine in ten patients were financed by it in 2012. That evidence highlights the central role of SUS, as the supporter of RRT in Brazil and as organizer of cooperation mechanisms on all sectors involved to improve the care provided to children with ESRD on chronic dialysis treatment [28].

We also found that one third of patients had their primary diagnosis unknown. This is a high proportion of ignorance of the underlying disease among children and adolescents in RRT when compared with other countries [5, 19]. Southeast region had the highest proportion of known primary diagnosis. This may be related to increased capacity of the dialysis centers located in that region to identify the underlying disease of ESRD.

Kidney transplantation is the modality of RRT of choice for children. Registry data from developed countries have shown that 65–80% of children and adolescents on RRT programs have a kidney transplant. The present study showed that one in four children on dialysis had previously received a kidney graft. These children, who return to dialysis after kidney transplant failure, usually accumulate two periods on dialysis (before and after the transplant) and a period of transplantation. Consequently, they present significant increase in morbidity associated with CKD, as immunological sensitization by raising antibodies against HLA antigens. This feature requires greater investment of resources and causes the clinical management of this sub-population is even more challenging [29].

The South region had a significantly higher proportion of these patients. This finding more probably reflects the existence of regional kidney transplantation reference centers and the better organization of local health services that work together to provide comprehensive and appropriate care for children with ESRD. In fact, we have detected that a large proportion of patients from the South region came from a center with high transplant activity [12].

In addition, the main known diagnosis was related to CAKUT. This finding is very similar to that evidenced by research in other countries that have identified CAKUT as the leading cause of ESRD [5, 19]. The prevalence of CAKUT was higher in the Southeast region and lower in the South region. Such difference should be associated with age and race, because the distribution of causes of pediatric ESRD varies according to them. Whereas CAKUT predominates in younger patients, glomerulonephritis is the leading cause in children older than 12 years of age [19]. Focal segmental glomerulosclerosis (FSGS) has shown to be three times more common in black than in white adolescents [30].

We found a longer time between primary diagnosis and initiation of chronic dialysis program in the group of children from the Southeast. This significant difference can be explained by two marked characteristics of that region: is the region with more proportionately known primary diagnosis and with the highest level of socioeconomic development, which enables the early management of CKD in a better equipped health system.

In this study, we estimated the prevalence and incidence of pediatric ESRD cases undergoing dialysis and, as a smaller number of children undergo kidney transplant, our figures underestimate the actual incidence and prevalence of pediatric ERSD under RRT. Thus, the results and comparisons should be considered with caution if one takes into account all RRT pediatric patients. On the other hand, due to operational difficulties and impossibilities of gathering and combining information from national transplant registries, several other studies, not only the present one, have evaluated just the pediatric population on chronic dialysis treatment [1].

Another limitation of the study is that even using a careful sample of the Brazilian dialysis centers, which was obtained from the best source available of national centers, the sample loss of dialysis centers and the inadvertently exclusion of some of them from the analysis, particularly those with more children under treatment may have occurred and influenced the precision of the incidence and prevalence estimates. Furthermore, grouping the North and Midwest regions for the implementation of the sampling plan may have partially affected the interpretation of the results, since these regions have some different socioeconomic characteristics.

Additionally, as this is a cross-sectional study we may not have detected some new or prevalent cases at the precise time when they started dialysis in a specific center included in the study, what could be better ascertained in a prospective study.

Moreover, it is worth noting that, although the study was performed under rigorous data collection and analysis criteria, it was not made direct contact with the children or their guardians, restricting access to available data in patients’ records. Particularly, the information about the children's age at primary diagnosis was just partially collected. Thus, the absence of these data may have interfered in some results presented.

On the other hand, this study is the only recent national representative data for Brazilian children related to chronic dialysis treatment. In addition, as desirable in studies based on demographic health surveys, we performed all statistical procedures with the expansion technique of complex sample to ensure the reliability of the estimates.

It is also noteworthy that our study was performed in a developing and continental country with a large territorial area and great socioeconomic and cultural diversity. That scenario conducted us to compare rates and epidemiological characteristics among regions. Thus, the estimation of the results gives us a comprehensive view of the epidemiology of ESRD in Brazilian children, which can be used for comparisons with findings from other countries with similar developmental characteristics.

## Conclusion

We conclude that Brazil has a low prevalence and incidence rates of pediatric ESRD cases undergoing chronic dialysis treatment. The South and Southeast regions showed to be the better prepared to deal with pediatric ESRD as they have the highest rates of children in RRT and the better features of management of children with CKD. However, there are still many gaps to be filled in the knowledge of the characteristics of Brazilian children with ESRD. The likely underdiagnosis of the causes of CKD, differences in access to healthcare and regional socioeconomic inequalities are the phenomena to be tackled by organs and health managers [31].

Therefore, to offer an integrated and complete assistance for children with ESRD on chronic dialysis treatment is a challenge that must be met by all Brazilian states. Only joint and well planned strategies for reducing inequities and adequacy of services may provide an appropriate care setting for this population group.

Since there is a wide regional diversity, and the number of patients in Brazil and worldwide is increasing [11], more studies are needed to improve the understanding of the epidemiological characteristics of pediatric ESRD and hence allow comparisons among countries to improve the quality of care provided.
